# Comparison of two targeted ultra-deep sequencing technologies for analysis of plasma circulating tumour DNA in endocrine-therapy-resistant breast cancer patients

**DOI:** 10.1007/s10549-021-06220-9

**Published:** 2021-06-07

**Authors:** Georgios Nteliopoulos, Karen Page, Allison Hills, Karen Howarth, Warren Emmett, Emma Green, Luke J. Martinson, Daniel Fernadez-Garcia, Robert Hastings, David S. Guttery, Laura Kenny, Justin Stebbing, Susan Cleator, Farah Rehman, Kelly L. T. Gleason, Andrijac Sanela, Charlotte Ion, Amelia J. Rushton, Nitzan Rosenfeld, R. Charles Coombes, Jacqueline A. Shaw

**Affiliations:** 1 Department of Surgery and Cancer, Division of Cancer, Imperial College London, London, UK; 2 Department of Genetics and Genome Biology and Leicester Cancer Research Centre, College of Life Sciences, University of Leicester, University Road, Leicester, LE1 7RH UK; 3 Inivata Ltd, Granta Park, Cambridge, UK; 4 Department of Medical Oncology, Imperial College London, Charing Cross Hospital, London, UK

**Keywords:** Metastatic breast cancer, Endocrine therapy resistance, Circulating tumour DNA (ctDNA), Next-generation sequencing

## Abstract

**Purpose:**

There is growing interest in the application of circulating tumour DNA (ctDNA) as a sensitive tool for monitoring tumour evolution and guiding targeted therapy in patients with cancer. However, robust comparisons of different platform technologies are still required. Here we compared the InVisionSeq™ ctDNA Assay with the Oncomine™ Breast cfDNA Assay to assess their concordance and feasibility for the detection of mutations in plasma at low (< 0.5%) variant allele fraction (VAF).

**Methods:**

Ninety-six plasma samples from 50 patients with estrogen receptor (ER)-positive metastatic breast cancer (mBC) were profiled using the InVision Assay. Results were compared to the Oncomine assay in 30 samples from 26 patients, where there was sufficient material and variants were covered by both assays. Longitudinal samples were analysed for 8 patients with endocrine resistance.

**Results:**

We detected alterations in 59/96 samples from 34/50 patients analysed with the InVision assay, most frequently affecting *ESR1, PIK3CA* and *TP53*. Complete or partial concordance was found in 28/30 samples analysed by both assays, and VAF values were highly correlated. Excellent concordance was found for most genes, and most discordant calls occurred at VAF < 1%. In longitudinal samples from progressing patients with endocrine resistance, we detected consistent alterations in sequential samples, most commonly in *ESR1* and *PIK3CA*.

**Conclusion:**

This study shows that both ultra-deep next-generation sequencing (NGS) technologies can detect genomic alternations even at low VAFs in plasma samples of mBC patients. The strong agreement of the technologies indicates sufficient reproducibility for clinical use as prognosic and predictive biomarker.

**Supplementary Information:**

The online version contains supplementary material available at 10.1007/s10549-021-06220-9.

## Introduction

Over the past few decades, therapies have been developed for the treatment of the most predominant (estrogen receptor (ER)-positive) subset of breast cancers (BC) and anti-estrogens (e.g. tamoxifen, fulvestrant) and aromatase inhibitors (e.g. letrozole, anastrozole, exemestane) are in widespread use as adjuvant and metastatic therapies [1].

However, many patients become resistant to treatment and research into the mechanisms of resistance has been hampered by the invasive approach of obtaining tissue biopsies from patients with endocrine-treatment-resistant metastatic disease (mBC). More recently, characterisation of mBC has been made more feasible by molecular profiling of circulating cell-free DNA derived from tumour cells [termed circulating tumour DNA (ctDNA)], through liquid biopsy [2, 3]. This ctDNA is released by dying cancer cells and active secretion and is considered to be more reflective of multiple metastatic subclones than tissue biopsy. Depending on the size, stage and activity of the cancer, tumour-specific alterations can be present in plasma samples in a background of cell-free DNA derived from healthy normal cells, with a variant allele fractions (VAFs) below 0.5% [4]. Therefore, there is a clinical need for the development, optimisation and validation of highly sensitive methods for detection of ctDNA.

To determine the optimum ctDNA-based approaches for guiding clinical treatment decisions, there is a need for inter-laboratory comparison of technologies used for ctDNA variant detection. There are multiple assays for ctDNA analysis, including those targeting a single/small number of variants, such as digital droplet-PCR (ddPCR) and bead-based digital PCR in emulsion (BEAMing), and broader targeted gene/mutation panels typically analysed by next-generation sequencing (NGS) [5]. To date, few comparisons have been published comparing different platforms for ctDNA measurement [6], most of which compare digital PCR (dPCR) with NGS platforms [7, 8].

This study focusses on the comparison of two different commercial NGS methods of ctDNA detection/quantification, the InVisionSeq™ ctDNA Assay, developed by Inivata Limited and the Oncomine™ Breast cfDNA Assay_v1 by Thermo Fisher Scientific. The InVision assay identifies single nucleotide variants (SNVs), insertions and deletions (INDELs) and copy number variants (CNVs) across whole genes and hotspots in a 36-gene panel [9]. Together with analysis of gene fusion events in plasma, it forms part of the InVisionFirst®-Lung comprehensive genomic profiling liquid biopsy assay that is in clinical use. The technology has demonstrated an excellent limit of detection (LOD) in analytical validation studies [9, 10], with 99.48% sensitivity for SNVs present at VAF range 0.25–0.33% and 56.25% sensitivity at VAF range 0.06–0.08% while retaining high specificity (99.9997% per base). High concordance with dPCR in patients with NSCLC was also demonstrated [10, 11]. The Oncomine™ Breast cfDNA Assay_v1 is a more focussed NGS assay which combines Ion AmpliSeq™ and Tag Sequencing technologies enabling detection of primary driver and resistance mutations (SNVs/INDELs) from ctDNA down to a level of 0.1% with ~ 81% sensitivity and 99.9% specificity [12].

In this report, we describe our direct comparison of both methods and show excellent correlation between both variants and VAFs detected by the two approaches. Further, we present results of targeted sequencing of ctDNA in a small group of patients with mBC, who had become resistant to endocrine therapy. We show excellent concordance between the two assays, in agreement with a recent study comparing 2 NGS platforms that found no significant differences in detection of alterations in plasma of patients with mBC [13]. These findings contrast with 2 previous studies, one that found low concordance when comparing plasma of prostate cancer patients in 2 platforms [14] and one finding substantial variability among the 4 ctDNA assays compared, with most discordance observed at VAF < 1% [15].

## Patients and methods

### Ethics statement

The study protocol was approved by the Riverside Research Ethics Committee (Imperial College Healthcare NHS Trust; REC reference number: 07/Q0401/20). Blood sample collection was conducted in accordance with the Declaration of Helsinki. All patients gave written informed consent before participation.

### Blood processing and extraction of total cfDNA

A total of 96 blood samples from 50 unselected patients with radiologically-confirmed mBC were collected into EDTA-containing tubes (BD Biosciences) and processed to plasma within 2 h of collection. Total cfDNA was extracted from 3 mL of plasma using the QIAamp Circulating Nucleic Acids Kit (Qiagen) and quantification of eluted cfDNA was performed using the Qubit® 2.0 dsDNA high sensitivity assay (Thermo Fisher Scientific) as described previously [16].

### Targeted deep sequencing

A minimum of 20 ng total cfDNA isolated from 35 plasma samples were sequenced using the two established technologies; the InVisionSeq™ ctDNA Assay (Inivata, Cambridge) v1.4 or v1.5 (Supplementary Tables S1, S2) and the Oncomine™ Breast cfDNA Assay_v1 (Supplementary Table S3) (Thermo Fisher Scientific, Waltham, MA). Due to limiting cfDNA, the remaining 61 plasma samples were analysed using the InVision assay only.

The InVision assay profiles a combination of SNVs and INDELs across 35 cancer genes (v1.4) or 36 genes (v1.5), as well as CNVs in 4 genes. The Oncomine assay analyses SNVs/INDELs covering > 150 hotspots in 10 frequently mutated genes in BC. Seven genes are overlapping and hotspot mutations present in both panels were compared (Supplementary Table S4). A more detailed explanation of sequencing analysis is provided in the Supplementary Methods.

### Statistical analysis

For each sample pair the VAF and number of detected alterations (NDA) were compared between the two platforms using the Mann–Whitney *U* test and the Wilcoxon matched-pairs signed-ranks test. Spearman’s rank correlation coefficient and linear regression were used to determine whether the variables were correlated. Agreement in terms of detected altered genes were explored through Cohen's kappa coefficient. Statistical analysis was performed using Prism GraphPad_v6, San Diego, CA).

### Analysis of sequential patient samples

Eight patients with ER-positive BC and proven distant metastases were included as a small sub-study, where samples were collected at the beginning, and/or at some point during, endocrine therapy for mBC, and again at the end of therapy when they were no longer responding. All 8 patients had received one or more additional lines of endocrine therapy prior to that undergone at the time of blood sampling. Total cfDNA levels, circulating tumour cells (CTCs) counts and protein markers serum cancer antigen 15–3 (CA15-3) and alkaline phosphatase (ALK-PHOS) were also measured (Supplementary Methods).

## Results

### InVisionSeq™ ctDNA assay

**Fig. 1:**
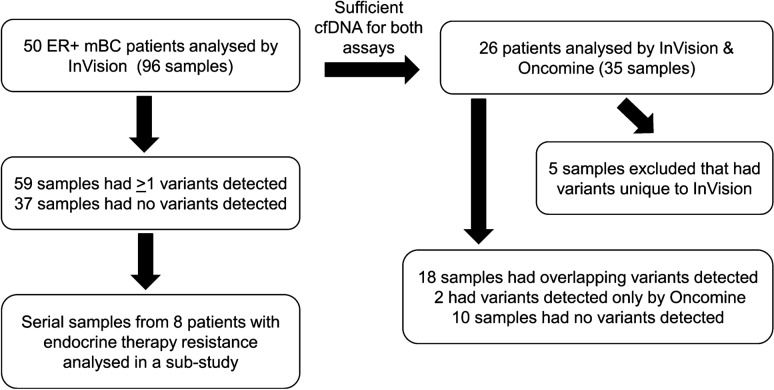
Diagram demonstrating the patients/samples used for each experiment: The numbers of patients, samples and variants detected are depicted

Ninety-six cfDNA samples from 50 patients were analysed with the InVisionSeq™ assay, of which 59 (61.5%) had 1 or more variants detected (Supplementary Table S5, Fig. 1). A total of 174 alterations in 20 genes were detected, 11 of which were amplifications in *FGFR1* (*n* = 9), *ERBB2* (*n* = 1) and *MET* (*n* = 1). SNVs comprised mainly missense but also truncating, splice-site and synonymous variants while INDELS comprised a mix of inframe and frameshift variants. VAFs ranged between 0.07 and 59.6%, with a median of 1.18%. Of 163 variants detected, 78 (47.9%) were detected at VAF ≤ 1%, and 25 (15.3%) were detected at VAF ≤ 0.25%. The most mutated genes were *ESR1* (*n* = 53), *TP53* (*n* = 32), *PIK3CA* (*n* = 31) and *ERBB2* (*n* = 10). As for occurrence of mutations in each patient, 34/50 patients (68%) had at least one mutation detected, with *PIK3CA* [19/50 (38%)], *ESR1* [17/50 (34%)] and *TP53* [16/50 (32%)] being the most frequent, followed by *ERBB2* (14%), *FGFR1* (12%) and *GATA3* (10%).

### High concordance between ctDNA variants and VAF detected using the InVisionSeq™ ctDNA assay and oncomine™ breast cfDNA assay

**Fig. 2:**
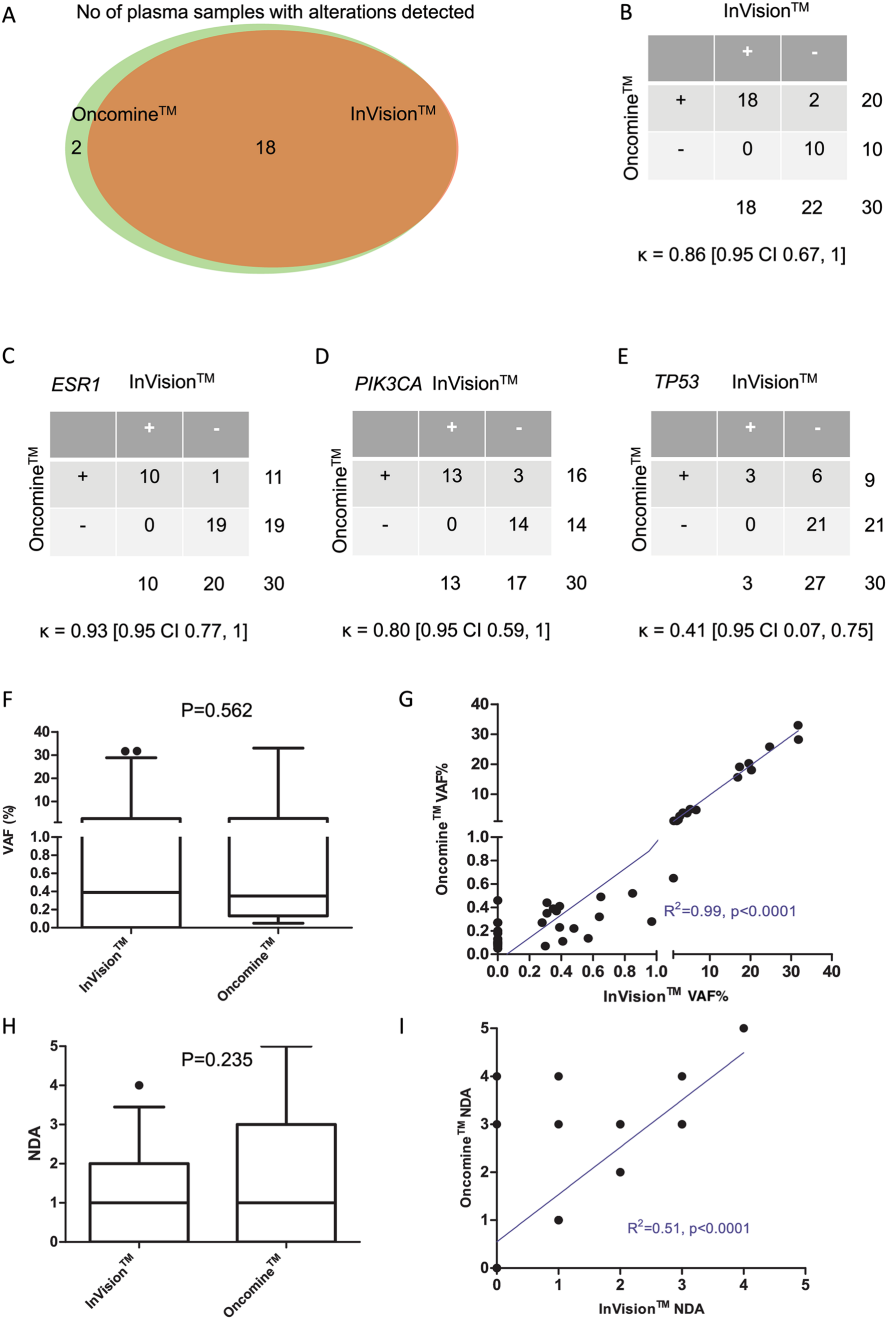
Comparison of the InVisionSeq™ assay v1.4 and the Oncomine™ Breast assay v1): Venn diagram demonstrating the number of plasma samples with detected alternations by the two ctDNA-targeted Next-Generation Sequencing platforms InVisionSeq™ and Oncomine™ (**a**). Comparison of the detected alterations overall (**b**) and in the most affected genes *ESR1* (**c**), *PIK3CA* (**d**) and *TP53* (**e**) and their respective Cohen’s Kappa coefficient. Paired variant allele fractions (VAF, **f**) and number of detected alterations (NDA, **h**) according to the analysis of the 2 NGS platforms. Mann–Whitney *U* test *p* values are demonstrated. Correlation analysis for VAF (**g**) and NDA (**i**) between the 2 NGS platforms. A linear regression model has been fitted with R^2^ and *p* value demonstrated

Overlapping variants in 7 genes (*AKT1, EGFR, ERBB2, ESR1, KRAS, PIK3CA* and *TP53*) covered by both Oncomine and InVision_v1.4 assays were compared in 30 of the 35 (85.7%) samples analysed with both assays (Table 1). Five plasma samples were excluded from analysis as all alterations detected were unique to the InVision assay. The results show high concordance between the 2 assays. 47 variants were identified by at least one platform; all 31 variants detected by the InVision assay were cofirmed by Oncomine, while 16 variants in 8 samples (VAF in the range 0.05–0.46%) affecting *TP53, PIK3CA* and *ESR1* were called only by Oncomine. Ten of 30 plasma samples (33.3%) had no variants detected by either assay. Twelve of 30 plasma samples (40%) had complete concordance with 1 or more alterations detected and 6/30 samples (20%) had partial concordance with 1 or more overlapping variants. Two of 30 samples (6.7%) showed no concordance that had variants detected by Oncomine only. Both were identified as below the minimum input DNA and one had low read depth during sequencing and were called as indeterminate for InVision_v1.4 (Fig. 2a, Table 1).

**Table 1:** Comaprison of overlapping variants detected with the InVisionSeq™ ctDNA assay (Inivata, v1.4) and Oncomine™ Breast cfDNA assay (Thermo Fisher Scientific, v1)

Patient number	External patient ID	Gene name	Protein change	Probability of being inherited	InVision VAF%	Oncomine VAF%	QC Flag	Depth flag
23	1333	ESR1	p.Y537S	Somatic	0.31	0.35	PASS	QS1
23	1333	PIK3CA	p.E545K	Somatic	17.29	19.13	PASS	QS1
23	1333	ESR1	p.D538G	Somatic	24.69	25.82	PASS	QS1
23	1395	TP53	p.Y220C	Somatic	0.00	0.05	PASS	QS2
23	1395	ESR1	p.Y537S	Somatic	0.30	0.07	PASS	QS2
23	1395	PIK3CA	p.E545K	Somatic	0.35	0.39	PASS	QS2
23	1395	ESR1	p.D538G	Somatic	0.48	0.22	PASS	QS2
23	1402	ESR1	p.D538G	Somatic	0.00	0.27	PASS	QS2
23	1402	ESR1	p.Y537S	Somatic	0.28	0.27	PASS	QS2
23	1402	PIK3CA	p.E545K	Somatic	0.31	0.44	PASS	QS2
28	1385	TP53	p.R280T	Somatic	0.00	0.11	PASS	QS1
28	1385	PIK3CA	p.H1047R	Somatic	0.64	0.32	PASS	QS1
28	1385	KRAS	p.G12C	Somatic	1.05	0.65	PASS	QS1
28	1385	TP53	p.P278R	Somatic	3.40	3.85	PASS	QS1
28	1385	KRAS	p.G12D	Somatic	4.40	3.71	PASS	QS1
40	1290	TP53	p.R175H	Somatic	0.39	0.23	PASS	QS1
40	1290	ESR1	p.Y537C	Somatic	0.39	0.41	PASS	QS1
40	1290	PIK3CA	p.H1047R	Somatic	31.66	33.01	PASS	QS1
48	1398	ESR1	p.Y537N	Somatic	31.77	28.22	PASS	QS2
59	1323	ESR1	p.D538G	Somatic	16.85	15.64	PASS	QS1
102	1319	PIK3CA	p.H1047R	Somatic	6.64	4.76	INDETERMINATE	QS3
102	1362	ESR1	p.Y537S	Somatic	0.85	0.52	INDETERMINATE	QS2
102	1362	PIK3CA	p.H1047R	Somatic	19.58	20.30	INDETERMINATE	QS2
125	1404	PIK3CA	p.E545K	Somatic	2.25	1.49	PASS	QS1
130	1310	No call for this sample			0.00	0.00	INDETERMINATE	QS2
130	1332	No call for this sample			0.00	0.00	INDETERMINATE	QS3
132	1244	No call for this sample			0.00	0.00	PASS	QS2
141	1320	No call for this sample			0.00	0.00	PASS	QS2
156	1393	No call for this sample			0.00	0.00	PASS	QS1
160	1307	ESR1	p.D538G	Somatic	2.64	2.74	INDETERMINATE	QS3
167	1309	PIK3CA	p.E545K	Somatic	0.00	0.08	INDETERMINATE	QS3
167	1309	PIK3CA	H1047R	Somatic	0.00	0.10	INDETERMINATE	QS3
167	1309	TP53	R248W	Somatic	0.00	0.13	INDETERMINATE	QS3
167	1309	TP53	G244C	Somatic	0.00	0.46	INDETERMINATE	QS3
174	1344	TP53	p.Y220C	Somatic	0.00	0.10	PASS	QS1
174	1344	ESR1	p.Y537N	Somatic	1.87	1.17	PASS	QS1
174	1344	PIK3CA	p.E542K	Somatic	5.18	5.04	PASS	QS1
182	1311	PIK3CA	p.E545Q	Somatic	0.57	0.14	INDETERMINATE	QS3
182	1311	ESR1	p.Y537S	Somatic	0.97	0.28	INDETERMINATE	QS3
183	1322	No call for this sample			0.00	0.00	INDETERMINATE	QS1
187	1391	PIK3CA	p.E545K	Somatic	0.65	0.49	PASS	QS1
188	1331	No call for this sample			0.00	0.00	INDETERMINATE	QS2
195	1356	No call for this sample			0.00	0.00	INDETERMINATE	QS2
196	1357	TP53	p.R282W	Somatic	0.00	0.09	PASS	QS1
196	1357	ESR1	p.Y537N	Somatic	0.00	0.20	PASS	QS1
196	1357	ESR1	p.D538G	Somatic	0.00	0.27	PASS	QS1
196	1357	PIK3CA	p.H1047R	Somatic	20.24	18.04	PASS	QS1
199	1361	TP53	p.G244D	Somatic	0.00	0.07	PASS	QS2
199	1361	PIK3CA	p.H1047R	Somatic	0.00	0.27	PASS	QS2
199	1361	KRAS	p.G12C	Somatic	0.37	0.37	PASS	QS2
200	1363	TP53	p.R175H	Somatic	0.00	0.09	INDETERMINATE	QS2
200	1363	PIK3CA	p.M1043V	Somatic	0.00	0.13	INDETERMINATE	QS2
200	1363	TP53	p.R175C	Somatic	0.00	0.18	INDETERMINATE	QS2
201	1364	No call for this sample			0.00	0.00	PASS	QS2
202	1366	No call for this sample			0.00	0.00	INDETERMINATE	QS2
226	1419	TP53	p.R282W	Somatic	0.41	0.11	PASS	QS1
228	1424	PIK3CA	p.H1047L	Somatic	1.15	1.12	PASS	QS1

Patient and serial sample numbers, mutation details (name of the affected gene, protein change and probability of being inherited) and the variant allele frequency (VAF) from both NGS platforms and the InVision depth and QC flags are depicted. The patients had serial samples, Patient 23 (1333, 1395, 1402), patient 102 (1319, 1362) and patient 130 (1310, 1332). QS3 indicates low read depth while indeterminate QC indicates DNA input below the validated input for the assay

The high congruence between the 2 platforms was also confirmed by excellent agreement across *ESR1* and *PIK3CA* (Fig. 2b–d), with moderate agreement for *TP53* (Fig. 2e). It is important to mention that due to limited plasma and resulting cfDNA for head-to-head comparison, unfortunately 7 samples had cfDNA input below the validated minimum for the InVision’s assay (and were considered indeterminate for InVision_v1.4) and therefore there is a risk of false-negative results for these samples.

In terms of VAF, of the 47 variants called, there were no statistically significant differences in the paired data (*p* = 0.662) or considered as a whole (*p* = 0.562) and high correlation was observed between the VAFs (Spearman *r* = 0.88 [0.99 CI 0.78, 0.93], *p* < 0.0001; Fig. 2f, g). The 16 variants with VAF ≥ 1% had the highest correlation (Spearman *r* = 0.98 [0.95 CI 0.94, 0.99], *p* < 0.0001), whereas the 31 variants with VAF < 1% had a lower correlation (Spearman *r* = 0.55 [0.95 CI 0.23, 0.71], *p* = 0.0013). As for the number of common detected alterations (NDAs), we found no significant differences when comparing as a whole (*p* = 0.235) in contrast to when comparing paired data (*p* = 0.013) whereas there was good correlation (Spearman *r* = 0.51 [0.95 CI 0.51, 0.87], *p* < 0.0001; Fig. 2h, i).

The alterations in genomic regions that are covered by only one assay are shown in Supplementary Table S6. Of those, 28 were represented only in the InVision panel whereas a single variant (*PIK3CA* p.Q546P) detected in 2 plasma samples was represented only by Oncomine (Supplementary Results).

### Longitudinal analysis of plasma ctDNA in patients receiving endocrine therapy

To determine the clinical utility of mutations detected in ER-positive mBC, 8 patients with ER + mBC were included as part of a sub-study. Therapies where ctDNA sequencing was assessed were anti-estrogens, one or more aromatase inhibitors, with or without chemotherapy (exemestane, palbociclib). Two patients received fulvestrant, 2 exemestane and everolimus, 2 palbociclib and letrozole, and the remaining 2 patients received a non-steroidal aromatase inhibitor (AZD4546).

All patients whose disease progressed showed an increase in ctDNA VAF, involving at least one of *PIK3CA* (*n* = 5), *GATA3* (*n* = 2) and/or *ESR1* (*n* = 5). Interestingly, all *PIK3CA* mutations involved residues 1047 or 545 and all *ESR1* mutations involved the 546–548 region, with two exceptions (p.544:L/−). Additional exon mutations were found in *TP53* (3 patients), *NFE2L2* (2 patients), *ERBB2, KRAS, GNAS, AKT1* (one patient). Amplifications of *FGFR1* or *MET* were detected in 2 patients. Several patients had polyclonal mutations in *ESR1* (*n* = 2), *TP53*, *ERBB2* or *GNAS* (*n* = 1).

**Fig. 3:**
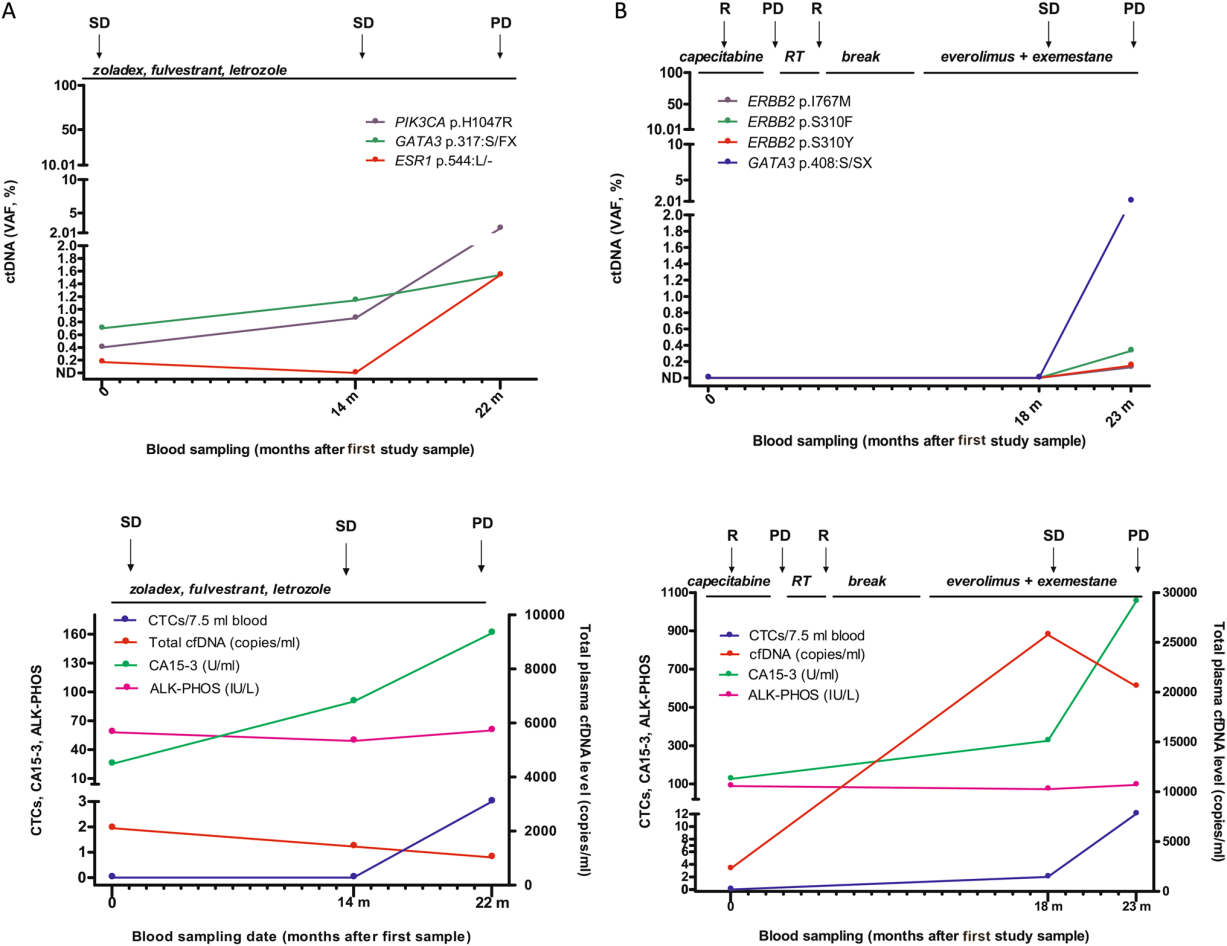
Serial monitoring of ctDNA and other blood biomarkers during endocrine therapy. Showing 2 patients with *ESR1, PIK3CA, GATA3* (**a**) and *ESR1, TP53* mutations (**b**) in ctDNA tracked during clinical progression. Variant allele fractions are shown as determined by sequencing. Bottom graphs show total cfDNA concentration (copies/mL), number of CTCs (per 7.5 mL of blood), CA15-3 (U/mL) and ALK-PHOS (IU/mL) for the same time points. Treatments details are given above each graph. *SD* stable disease, *PD* progressing disease, *VAF* variant allele frequency, *CTC* circulating tumour cell, *CA15-3* cancer antigen 15–3, *ALK-PHOS* alkaline phosphatase

Patient 1 (Pt.43; Fig. 3a) was diagnosed with ER + /PR + /HER2− invasive ductal carcinoma (IDC) with spinal metastasis. She had received Fluorouracil, epirubicin, cyclophosphamide and docetaxel (FEC-T) chemotherapy, tamoxifen, next zoladex followed by addition of fulvestrant and letrozole after worsening of liver metastases. The first research blood sample was collected while she was undergoing triple endocrine blockade, cfDNA sequencing revealed mutations in *ESR1* (p.544:L/ −), *GATA3* (p.317:S/FX) and *PIK3CA* (p.H1047R) at VAFs of 0.17%, 0.7% and 0.4%, respectively. The second research blood sample was collected 14 months later while her disease was stable. The *ESR1* mutation was absent, however, the mutant VAF increased for both *GATA3* (1.14%) and *PIK3CA* (0.86%). The patient remained stable before progressing with worsening of liver, brain and bone metastases, when *GATA3* and *PIK3CA* mutations were detected at higher levels again and *ESR1* p.544:L/- reappeared at 1.54%. As for the other markers, CA15-3 was increasing whereas CTCs were less informative and only detected at the last timepoint.

Patient 2 (Pt.73; Fig. 3b) was diagnosed with ER + /PR-/HER2 + IDC. She had received adjuvant tamoxifen therapy, as well as zoladex and eribulin prior to collection of the first blood sample. At this time, she began letrozole therapy but progressed with bone metastases 8 months later, when the second blood sample was collected. At that time, 2 *ESR1* mutations (p.Y537C and p.D538G) were detected at VAFs of 0.16% and 1.57% respectively. The patient was enrolled into a *FGFR* inhibitor clinical trial for a short time before progressing once again. Despite being switched to palbociclib (with letrozole), she progressed again 2 months before the third research sample when p.Y537C and p.D538G persisted and an additional *ESR1* mutation and a *TP53* mutation appeared. Samples 4 and 5, were taken 1 and 2 months later at a time of further bone progression, showing *ESR1* p.D538G and p.Y537S mutation at increased VAFs, an absence of p.Y537C and an emergence of a new *ESR1* mutation (p.L536H) in the final sample. The patient was switched to gemcitabine and carboplatin chemotherapy, however she died 3 months after the last sample was taken. All the other markers were also increasing during disease progression.

Data for the remaining 6 patients are shown in the Supplementary Material (Supplementary Figure S1).

## Discussion

This study focusses on comparison of two different NGS methods of ctDNA profiling; the InVision liquid biopsy platform, which utilizes eTAm-Seq® technology for the identification of low frequency mutations using a primer design strategy that allows for amplification of highly fragmented DNA, typical of ctDNA [9, 10]; and the Oncomine Breast Cancer panel, which is a BC-specific panel, which usesTag Sequencing technology to achieve low LOD of 0.1% for SNVs/short indels. Previous studies that used NGS platforms to compare ctDNA with tumour tissue DNA [17–19] demonstrated limited concordance, probably due to low tumour content in plasma and/or tumour heterogeneity.

More recent studies have compared NGS technologies for ctDNA analysis. Guardant360™ and PlasmaSELECT were compared in plasma from prostate cancer patients and showed lack of concordance in nearly 50% of patients [14]. However, these findings have been challenged, highlighting the lack of variant type or VAFs reporting and the discordance of low DNA copy numbers due to stochasticity [20, 21]. Two studies reanalysed the original data and found that indeed the alterations compared were mostly low-VAF and below the specified achievable LOD, whereas some germline variants were excluded only in one assay [22, 23]. Additionally, the cohort used was too indolent thus inappropriate for ctDNA testing [24]. In another study investigating ctDNA assay discordance, baseline plasma samples from 24 early-stage cancer patients were sent to 4 ctDNA sequencing vendors and compared with independently tested, time-matched, tumour-normal tissue pairs [15]. This orthogonal approach revealed substantial variability among the ctDNA assays with 58% discordance between matching tumour or other plasma sample, mostly observed for VAF < 1% but not > 10%, suggesting that most NGS assay discordance reflects technical variations rather than biological factors. Similarly, the Avenio ctDNA_Expanded panel and QIAseq Human_Comprehensive_Cancer panel were compared and demonstrated high coverage, sensitivity and concordance for the detection of clinically relevant variants but discordance at VAFs < 1% [25].

Our study demonstrates a high correlation between the VAFs detected, especially for variants with VAF > 1%, in agreement with Gerratana et al*.* who compared the NGS platforms PredicinePLUS™ and Guardant360™ in mBC [13]. As for lower VAFs, we must consider the LOD of each assay and the potential low DNA copy numbers. Although the Oncomine and the InVision assays report a LOD of 0.1% and 0.25%, respectively, Oncomine called 7 variants below the LOD (VAFs 0.05–0.09%) and InVision called a variant with VAF 0.18% in the overlapping region. In addition, 24 samples analysed only by InVision had mutations with a VAF below the LOD. Interestingly, of the 16 variants called only by Oncomine, 12 had VAFs below the LOD of the InVision assay (VAFs 0.05–0.24%). The variant *TP53* p.G244C with VAF 0.46% was called by Oncomine only, however Inivata reported low copy numbers of DNA molecules for this plasma sample. As additional plasma was not available to confirm discordant variants with an orthogonal method (dPCR), it is not possible to determine whether the additional Oncomine variants represent Oncomine false-positives, InVision false-negatives or both. However, in one instance (pt.196) where Oncomine detected a *TP53* mutation missed by the InVision assay (p.R282W; 0.09% VAF), InVision detected other *TP53* variants at higher levels in regions not covered by Oncomine (p.251:I/X 24.1% VAF, p.V157I 4.42% VAF) indicating that the Oncomine variants could be subclonal. The Oncomine assay has called variants as low as 0.05% in another study [26] while InVision has reported detection of 88.9% of SNVs at the VAF range of 0.13–0.16% and 56.3% at 0.06–0.08% [10].

Our study fulfils all the ctDNA test concordance study design criteria [27], since the paired-sample collection was concurrent and from BC samples at progression, while the VAFs were within the detection range of both tests. Comparison between the 2 technologies demonstrated a high agreement with only 2 of 30 (6.66%) discordant samples (in both of which Inivata reported as having low input DNA), suggesting sufficient reproducibility for clinical use. When comparing the assays across the most frequently mutated genes a high agreement was observed for *ESR1* and *PIK3CA* with low discordance rates (3.3% and 10%, respectively), comparable to other studies [13, 28] but only a moderate agreement for *TP53* (20% discordance rate). Similarly, robust concordance between tissue and blood was found for *PIK3CA* but not *TP53* mutation [29]. The majority of discordant calls occurred at VAF < 1% suggesting that detection of low-frequency mutations using NGS technologies could be limited by pre-analytical variables such as limited plasma volume, DNA isolation techniques and by sequencing artefactual errors, background noise, bioinformatics filtering thresholds and germline variant calls, resulting in false-positive discovery [15, 30]. Apart from technical reasons, biological factors such as clonal haematopoiesis of undetermined potential (CHIP)-mutations that lead to clonal expansion but not haematological neoplasia [31]- may also play a role. The detection of clonal haematopoiesis associated with *KRAS, TP53*, *JAK2* mutations has been reported in advanced-stage NSCLC [32], so some of the low-level *TP53* variants in our study might be CHIP-related.

Another part of the study was to investigate the utility of the InVision platform in mBC, since it has already proven utility in NSCLC [33–35]. Although the overlap of Oncomine and InVision was small (7 genes included in both assays), which is expected since InVision was designed for NSCLC, amongst those overlapping genes were genes frequently altered or implicated in the pathogenesis of mBC (*PIK3CA, TP53, ESR1*, *ERBB2*) [3]. Our results, similar to Fribbens et al. [36], demonstrate that the panel is fit-for-purpose for monitoring BC patients in a metastatic setting, since genetic alternations in many mBC-related genes were found in almost two thirds of patients. Somatic alternations were found in 20 genes and were mainly SNVs/INDELs but also few CNVs. *PIK3CA, ESR1* and *TP53* were the most affected genes, comparable with other studies that have used the InVision [36], Oncomine Breast Cancer [26], and PredicinePLUS™ [13] panels for hormone receptor-positive mBC.

The last part of our study was to follow mBC patients that show resistance to endocrine therapy, by analysis of longitudinal samples. There have been very few publications detailing the frequency of driver mutations in patients who have become resistant to multiple lines of endocrine therapy. Here we describe the frequent occurrence of *ESR1* and *PIK3CA* mutations in this clinical situation. Our study may have missed other important driver genes, however, based on the panel coverage. Very low-VAF *ESR1* mutations have been found in primary BC, and high-VAF in mBC, suggesting that *ESR1*-mutant clones are enriched by endocrine therapy [37]. A comprehensive survey of driver mutations in mBC demonstrated that the most frequent mutations associated with endocrine therapy-failure were found not only in *ESR1* but also in *ERBB2* and *NF1* when comparing metastases with primary tumours; they found that *ESR1* and *ERBB2*, *NF1* mutations (that activate MAPKinase signalling) were mutually exclusive. The third category of mutations found in endocrine-resistant patients were the transcription factors *MYC, FOXA1*, *TBX* genes (9%), also mutually exclusive with the *ESR1* (18%) and MAPKinase pathway (13%) mutations. Thus, in 60% of tumours detected mutations did not correspond to those known molecular mechanisms [38]. As regards other mutations associated with endocrine resistance in our study, *KRAS* [36], *AKT1* [39], *GATA3* [40, 41] and *GNAS* [21] were mutated in mBC.

One of the most interesting aspects of this study is the finding of multiple *ESR1* mutations. In case new therapeutics to delay/abolish the emergence of resistance were needed, then a focus on the 536–538 codons of *ESR1* and the 1047 and 545 codons of *PIK3CA* could be achieved by simpler/cheaper technologies including ddPCR, with some loss of sensitivity through splitting samples [36]. If inhibitors abrogating these constitutively active mutant proteins were discovered, longer remissions could be expected. However, in many patients, multiple mutations of *ESR1* seem to be present, implying the need for combination therapy.

The emergence of mutations seems to coincide with an increase in other markers (CTCs, CA15-3 and ALK-PHOS) in our study, however in some cases mutations appeared before the increase of the other markers. Previously, we demonstrated that CTCs and cfDNA are more informative than the conventional biomarkers (CA15-3 and ALK-PHOS) for prediction of overall survival in mBC [42], while we and others have shown that metastatic relapse was predicted with a lead-time of up 8.9 months [2] and 6.7 months [36] by utilizing ctDNA analysis [43].

The current study has limitations (small sample size, limited material available in some cases), however it shows encouraging results on the reproducibility of 2 ctDNA-based assays performed on samples from patients in a real-world setting [44]. Our data suggest that plasma-based ctDNA sequencing seems to be reliable with both technologies tested and could be used clinically, but clinicians need to be aware of technical limitations. These platforms can be used as prognostic biomarkers for detecting molecular relapse ahead of clinical or radiologic relapse and as predictive biomarkers for predicting drug resistance and facilitate potential change of therapy.

## Supplementary Information

Below is the link to the electronic supplementary material.

Supplementary file1 (PDF 616 kb)

Supplementary file2 (XLSX 53 kb)
