# Supplementary material for: Comparison of two targeted ultra-deep sequencing technologies for analysis of plasma circulating tumour DNA in endocrine-therapy-resistant breast cancer patients
Source: Breast Cancer Res Treat. 2021 Jun 7;188(2):465–76. doi: 10.1007/s10549-021-06220-9 (PMC8260509; doi:10.1007/s10549-021-06220-9)
Supplement: Supplementary file 1 — Supplementary file1 (PDF 616 kb) [file 10549_2021_6220_MOESM1_ESM.pdf]

SUPPLEMENTARY MATERIAL for manuscript entitled ‘Comparison of two targeted ultra-deep sequencing technologies for analysis of plasma circulating tumour DNA in endocrine-therapy-resistant Breast Cancer patients’ submitted to Breast Cancer Research and Treatment

Georgios Nteliopoulos[1*], Karen Page[2*], Allison Hills[1*], Karen Howarth[3], Warren Emmett[3], Emma Green[3], Luke J. Martinson[2], Daniel Fernadez-Garcia[2], Robert Hastings[2], David S. Guttery[2], Laura Kenny[4], Justin Stebbing[4], Susan Cleator[4], Farah Rehman[4], Kelly L.T. Gleason[4], Andrijac Sanela[4], Charlotte Ion[4], Amelia J. Rushton[1], Nitzan Rosenfeld[3], R. Charles Coombes[1] and Jacqueline A. Shaw[2]

1. Department of Surgery and Cancer, Division of Cancer, Imperial College London, London, UK
2. Department of Genetics and Genome Biology and Leicester Cancer Research Centre, University of Leicester, Leicester, UK
3. Inivata Ltd, Granta Park, Cambridge, UK
4. Department of Medical Oncology, Imperial College London, Charing Cross Hospital, London, UK

(*) GN, KP and AH contributed equally to this work

Corresponding Author: Jacqueline A. Shaw
Email: js39@le.ac.uk
Head Department of Genetics and Genome Biology, College of Life Sciences, University of Leicester, University Road, LEICESTER, LE1 7RH, UK
Orchid ID: https://orcid.org/0000-0003-4227-503X

Keywords: Metastatic breast cancer, endocrine-therapy resistance, Circulating Tumour DNA (ctDNA), Next-generation sequencing

Supplementary Methods

Blood processing and extraction of total cfDNA

20ml blood samples were collected from 50 radiologically confirmed MBC patients attending the Breast Clinic at Charing Cross Hospital in London. Patients were sampled at different times throughout the course of their treatment, aligning with their scheduled clinical appointments. Samples were taken along with concomitant clinical examination, and biochemical measurements, including serum cancer antigen 15-3 (CA15-3) and alkaline phosphatase (ALK-PHOS).

Targeted deep sequencing

Stored plasma was shipped on dry ice to the 2 laboratories in Cambridge and Leicester for independent analysis with the Inivata and Thermofisher workflows respectively. A minimum of 20ng total cfDNA isolated from 35 patients plasma samples were sequenced in duplicate, using the two established technologies; the InVisionSeq™ ctDNA Assay (Inivata, Cambridge) v1.4 or v1.5 (155 amplicons, 353 hotspots; Supplementary Tables 1, 2) and the Oncomine™ Breast cfDNA Assay v1 (26 amplicons, 155 hotspots; Supplementary Table 3) (Thermofisher, Leicester). The remaining 61 plasma samples were analysed using the InVisionSeq™ ctDNA Assay only.

In brief, the InVision liquid biopsy panels (v1.4/v1.5) were used for sequencing 35/36 cancer-related genes using gene specific primers designed to hotspots and entire coding regions of interest. NGS libraries were prepared using a two-step amplification process, incorporating replicate and patient specific barcodes and Illumina sequencing adaptors. Pooled libraries were quantified using Kapa Library Quantification Kit, and 1.8pM libraries analysed on an Illumina NextSeq 500 (300 cycle PE). Sequencing files were analysed using the Inivata Somatic Mutation Analysis (ISoMA) analytical pipeline (V1.15-1.17), and sequencing reads were clipped, merged and aligned. Coding and splice-site mutations in SNVs and Indels were annotated using Variant Effect Predictor (VEP) using the canonical transcript for each gene.

For the Oncomine™ Breast cfDNA Assay, library preparation was performed according to the manufacturer's instructions. In brief, targets were amplified from cfDNA, the target amplicons were purified and amplified with barcoded primers, the barcoded libraries were purified, size selected and quantified. Templating was performed on the Ion Chef using the Ion 530 Kit-Chef

and 200bp-sequencing was performed using the Ion-Torrent-S5 on Ion 530 Chip (Thermo Fisher Scientific). 12 samples were multiplexed aiming for mean depth coverage on each chip, aiming for mean molecular coverage of 2500, allowing for detecting of mutations at variant allele fraction (VAF) 0.1%. Base calling, mapping and alignment were performed using Torrent-Suite-v5 software, with overall coverage and variants were called using the coverageAnalysis v5.2 and variantCaller v.5.2 plugins, respectively, with default analysis parameters.

For a full list of all genes, type of genetic alternations and the exon where the alternation is located targeted by each approach see Supplementary Table S4.

Analysis of sequential patient samples

Further, clinical and imaging assessment data detailing response to therapy was available for all 8 patients, as well as biochemical assessment measures (liver function tests, circulating tumour cell - CTC counts and CA15-3 levels) prior to, and at the time of, blood sampling. Detection, enrichment and enumeration of CTCs of epithelial origin were performed using the CELLSEARCH® system and the CELLSEARCH® Epithelial Cell Kit (Menarini Silicon Biosystems). Briefly, 7.5 ml of blood were collected in CellSave tubes (Menarini Silicon Biosystems), immunomagnetic capture of CTCs based on EpCAM and cell labelling were performed on the CELLTRACKS®AUTOPREP® System and detection of CTCs based on morphological characteristics, positive expression of cytokeratins (8, 18, 19) and absence of the leukocyte marker CD45 were performed using the CELLTRACKS ANALYZER II® System. MBC samples with ≥5 CTCs were considered positive.

Supplementary results

High concordance between ctDNA variants and variant allele fraction detected using the InVisionSeq[TM] ctDNA Assay and Oncomine[TM] Breast cfDNA Assay

If we consider only the overlapping samples and include all the alternations called by the InVision panel a total of 59 variants were identified in 16 genes including missense (n=46), frameshift (n=3), synonymous (n=3), truncating mutations (n=3), splice variants (n=1) and amplifications (n=3). The VAFs, reflected by 56 SNVs and INDELs, detected in ctDNA ranged from 0.18 to 59.61%, median VAF: 2.54%. Of these 59 variants, 28 were not represented on the Oncomine[TM] panel (Supplementary Table 5). All the remaining 31 variants (100%) detected by InVisionSeq[TM] were called by Oncomine[TM]. A single variant (*PIK3CA* p.Q546P) was detected by Oncomine[TM] in 2 plasma samples (VAFs 0.09% and 0.42%), that was not represented by the by InVisionSeq[TM] ctDNA assay. Oncomine[TM] also detected an additional 16 variants in 8 samples (0.05-0.46% VAF) in 3 genes (*TP53*, *PIK3CA* and *ESR1*) that were represented by, but not detected, by the InVision First[TM] First[TM] assay. If we consider all the variants identified in ctDNA by Oncomine[TM] (n=49), the median VAF was 0.35 and the range from 0.05 to 31.05%.

Longitudinal analysis of sequential patient samples on endocrine therapy

Patient 3 (Pt.56; Figure S1A) was diagnosed with ER+/PR+/HER2- IDC in May 2006, commenced neo-adjuvant chemotherapy. The patient relapsed in February 2012 with bone, liver and lung metastases, and was responding to capecitabine therapy at the time of collection of the first research blood sample, in which no mutations were detected in the plasma. One year later, endocrine therapy with everolimus and exemestane was started, on which she remained stable for 6 months (no mutations detected at this time point but total levels of ctDNA were elevated 10-fold. However, the patients started progressing with new liver metastases and five months later was sampled again and a *GATA3* p.408:S/SX frameshift mutation was detected at 2.06% VAF, alongside three low-level *ERBB2* missense variants; p.S310Y (0.15%) p.S310F (0.33%) and p.I767M (0.13%) and a *FGFR1* amplification. The patient died 2 months after the last sample. CTC number and CA15-3 were elevated mostly at the last time point while total cfDNA levels fell by 20%.

Patient 4 (Pt.174; Figure S1B) was diagnosed with ER+/PR+/HER2- IDC 2011 and received letrozole, which she progressed on and was switched to exemestane in 2013. She had been

receiving exemestane for 9 months and fulvestrant for 3 months prior to collection of the first research blood sample. At this point (stable disease), 4 mutations were detected; a most likely germline *NFE2L2* p.R43W at a VAF of 50.85%, a *PIK3CA* p.E542K at 2.90%, a synonymous *GNAS* at 0.64% and an *ESR1* p.Y537N at 0.34%. The patient gradually progressed despite this treatment and developed skull metastases. After 5 months *NFE2L2* remained at 53.11% VAF, *PIK3CA* and *ESR1* increased VAF and *GNAS* was disappeared, although a different synonymous *GNAS* mutation appeared at 0.18%. CTC number and CA15-3 were elevated, in contrast to cfDNA levels and ALK-PHOS.

Patient 5 (Pt.102; Figure S1C) was initially diagnosed with ER+/PR-/HER2+ IDC in 1996. In February 2007 she started progressing and spent some time under anastrazole therapy, before changing to exemestane, and back to anastrazole, before progressing again in 2009 with spinal metastases. She started exemestane and everolimus and fulvestrant in March 2014 and after 8 months the first sample was taken, when common *TP53* (0.26% VAF) and *PIK3CA* (6.64% VAF) mutations were detected. She then progressed on endocrine therapy 3m later, just before the second research blood sample. At sample 2 an *ESR1* mutation, Y537S, appeared, the frequency of *PIK3CA* increased to 19.58% and *TP53* p.H178P was no longer detectable. All other markers were slightly increased.

Patient 6 (Pt.125; Figure S1E) was diagnosed in 2009 with ER+/PR+/HER2- ILC. She had letrozole and tamoxifen, before relapsing in 2013 with bone metastasis. She had been receiving anastrozole since August 2013 when the first research blood was taken and remained stable on it until May 2016 when she progressed. *PIK3CA* p.E545K increased and *KRAS* p.G12D appeared prior to the detection of progression by imaging. Both the number of CTCs and CA15-3, ALK-PHOS levels also increased prior to the detection of progression by imaging.

Patient 7 (Pt.113; Figure S1E) was initially diagnosed with ER+/PR-/HER2+ invasive lobular carcinoma (ILC) in 1993 and underwent tamoxifen therapy for 7 years. She recurred in 2004 and relapsed further, with bone metastasis, in May 2012. She received everolimus and exemestane treatment for 2 years, and eribulin for 4 months, prior to enrolment in our research study. Five blood samples were taken over 3 months, while the patient was steadily progressing with brain and leptomeningeal metastases, receiving radiotherapy and methotrexate. Multiple *ESR1* mutations on the 536-538 regions were present at the time of the first sample (p.Y537S at VAF of 15.79% and 4 at low level with VAF<1%), a *PIK3CA* p.E545K and a *NFEL2* p.E82A at low level too. All mutations levels were reduced during whole brain radiotherapy (the low levels ones disappeared) but were increased again after the start

of methotrexate, while a *FGFR1* amplification appeared after sample 2. Number of CTCs was increasing before a decrease at the fifth sample and CA15-3 increased before a big drop at the fourth sample.

Patient 8 (Pt.200; Figure S1F) was diagnosed with ER+/PR+/HER2- IDC in 2014 and received adjuvant tamoxifen. She progressed in January 2015 and underwent treatment with zoladex and letrozole. She was stable when the first sample was taken in March 2015, when no mutations were detected. Then she slowly progressed again, with bone metastases detected by imaging, in September 2015. At this point 5 SNVs were detected (3 *TP53* mutations, a *PIK3CA* p.E545K and an *AKT1* p.E17K and one *MET* amplification while total cfDNA levels fell by ~7-fold.

Relationship to other markers of disease activity

We also measured levels of other blood-based markers at the same time points as the ctDNA sequencing: in all cases, at least two measures of disease activity were worse (levels increased), with the exception of patient 182 where only one measure was worse (total cfDNA level). All except one patient, patient 43 who had no CTCs at any time point, had measurable CTCs, and in all except patient 182 these showed a rise in number (7 of 8 cases; 87.5%).

Supplementary Tables

Supplementary Tables S1, S2, S3 and S5 are attached separately as excel files

Supplementary Table S4: List of all genes targeted by each panel. Type of genetic alternations (SNVs, INDELs, CNVs, Fusions) and the exon where the alternation is located also demonstrated

	InVisionFirst™ ctDNA Assay v1.5							InVisionFirst™ ctDNA Assay 1.4						Oncomine™ Breast cfDNA Assay v1				common
	GENE	SNVs	INDELS	CNVS	FUSIONS	EXONS		GENE	SNVs	INDELS	CNVS	EXONS		GENE	SNVs	INDELS	EXONS	
1	**AKT1**	Y	Y	N	N	3	1	**AKT1**	Y	Y	N	4	1	**AKT1**	Y	Y	4	1
2	**ALK**	Y	Y	N	**Y**	22, 23, 25	2	**ALK**	Y	Y	N	23, 24, 25						
3	**BRAF**	Y	Y	N	N	11, 15	3	**BRAF**	Y	Y	N	11, 15						
4	**CCND1**	Y	Y	N	N	1	4	**CCND1**	Y	Y	N	1						
5	**CDKN2A**	Y	Y	N	N	1, 2, 3	5	**CDKN2A**	Y	Y	N	1-3						
6	**CTNNB1**	Y	Y	N	N	3	6	**CTNNB1**	Y	Y	N	3						
7	**EGFR**	Y	Y	Y	N	7, 15, 18, 19, 20, and 21	7	**EGFR**	Y	Y	Y	7,15, 18-21, amplifications	2	**EGFR**	Y	Y	21	2
8	**ERBB2**	Y	Y	Y	N	8, 17, 19, 20, 21	8	**ERBB2**	Y	Y	Y	8, 17, 19, 20, amplifications	3	**ERBB2**	Y	Y	20	3
													4	**ERBB3**	Y	Y	4, 8, 9, 10	
9	**ESR1**	Y	Y	N	N	4, 8	9	**ESR1**	Y	Y	N	4, 8	5	**ESR1**	Y	Y	6, 8, 9	4
													6	**FBXW7**	Y	Y	11	
10	**FGFR1**	N	N	Y	N		10	**FGFR1**	N	N	Y	amplifications						
11	**FGFR2**	Y	Y	N	N	7, 9, 12, 14	11	**FGFR2**	Y	Y	N	5, 7, 10, 12						
12	**FGFR3**	Y	Y	N	N	7, 9, 14	12	**FGFR3**	Y	Y	N	7, 10						
							13	**FOXL2**	Y	Y	N	1						
13	**GATA3**	Y	Y	N	N	4, 5, 6	14	**GATA3**	Y	Y	N	5, 6						
14	**GNA11**	Y	Y	N	N	5	15	**GNA11**	Y	Y	N	5						
15	**GNAQ**	Y	Y	N	N	5	16	**GNAQ**	Y	Y	N	5						
16	**GNAS**	Y	Y	N	N	8	17	**GNAS**	Y	Y	N	8						
17	**HRAS**	Y	Y	N	N	2, 3	18	**HRAS**	Y	Y	N	3, 4						
18	**IDH1**	Y	Y	N	N	4	19	**IDH1**	Y	Y	N	4						
19	**IDH2**	Y	Y	N	N	4	20	**IDH2**	Y	Y	N	4						
20	**KIT**	Y	Y	N	N	9, 11, 17	21	**KIT**	Y	Y	N	9, 11						
21	**KRAS**	Y	Y	N	N	2, 3, 4	22	**KRAS**	Y	Y	N	2, 3	7	**KRAS**	Y	Y	3	5
22	**MAP2K1**	Y	Y	N	N	2, 3, 6												
							23	**MED12**	Y	Y	Y	2						
23	**MET**	Y	Y	Y	N	7, 14, 19, 20	24	**MET**	Y	Y	N	14, amplifications						
24	**MYC**	Y	Y	N	N	2, 3,	25	**MYC**	Y	Y	N	2,3						
25	**NFE2L2**	Y	Y	N	N	2	26	**NFE2L2**	Y	Y	N	2						
26	**NRAS**	Y	Y	N	N	2, 3	27	**NRAS**	Y	Y	N	3, 4						
27	**NTRK1**	Y	Y	N	N	14, 15												
28	**NTRK3**	Y	Y	N	N	16												
29	**PDGFRA**	Y	Y	N	N	18	28	**PDGFRA**	Y	Y	N	12, 14, 18						
30	**PIK3CA**	Y	Y	N	N	2, 10, 21	29	**PIK3CA**	Y	Y	N	2, 8, 10, 21	8	**PIK3CA**	Y	Y	5, 8, 10, 21	6
31	**PPP2R1A**	Y	Y	N	N	5, 6	30	**PPP2R1A**	Y	Y	N	5, 6						
32	**PTEN**	Y	Y	N	N	1, 2, 4, 5, 6, 7, 8, 9	31	**PTEN**	Y	Y	N	1, 2, 4-9						
							32	**RET**	Y	Y	N	11, 16						
33	**ROS1**	Y	Y	N	**Y**	37, 38												
													9	**SF3B1**	Y	Y	16	
34	**STK11**	Y	Y	N	N	1 through 9	33	**STK11**	Y	Y	N	1-9						
35	**TP53**	Y	Y	N	N	1 through 11	34	**TP53**	Y	Y	N	1-11	10	**TP53**	Y	Y	5, 7, 8, 9	7
36	**U2AF1**	Y	Y	N	N	2, 6	35	**U2AF1**	Y	Y	N	2,6						

Supplementary Table S6: List of non-overlapping variants covered by both_InVision First^{TM} ctDNA assay (Inivata, v1.4) and Oncomine™ Breast cfDNA assay (Thermo Fisher Scientific, v1). Patient and sample numbers, the mutation details (name of the affected gene, protein change, consequence and probability of being inherited) and the variant allele fraction (VAF) from both NGS platforms. Not on panel indicates that the panel does not cover the variants

Patient No	Sample No	Gene name	Protein change	Consequence	Probability of being inherited	InVision VAF%	Oncomine VAF%
40	1290	TP53	p.I195F	missense_variant	Somatic	0.33	not on panel
175	1297	FGFR3	-	splice_donor_variant	Somatic	14.08	not on panel
175	1297	FGFR2	p.N550K	missense_variant	Somatic	44.67	not on panel
175	1297	STK11	p.D350D	synonymous_variant	Potentially inherited	59.61	not on panel
167	*1309*	*PIK3CA*	*p.Q546P*	*missense_variant*	*Somatic*	not on panel	0.42
182	1311	TP53	p.E51*	stop_gained	Somatic	2.44	not on panel
102	1319	TP53	p.H178P	missense_variant	Somatic	0.26	not on panel
59	1323	GATA3	p.408-409:-/X	frameshift_variant	Somatic	9.65	not on panel
59	1323	FGFR1	NA	amplification		NA	not on panel
174	1344	GNAS	p.L846L	synonymous_variant	Somatic	0.18	not on panel
174	1344	NFE2L2	p.R43W	missense_variant	Potentially inherited	53.11	not on panel
196	1357	TP53	p.V157I	missense_variant	Somatic	4.42	not on panel
196	1357	ERBB2	p.S310Y	missense_variant	Somatic	14.02	not on panel
196	1357	TP53	p.251:I/X	frameshift_variant	Somatic	24.14	not on panel
197	1358	PTEN	p.297-298:-/X	frameshift_variant	Somatic	15.73	not on panel
197	1358	TP53	p.Q192*	stop_gained	Somatic	19.39	not on panel
12	1368	STK11	p.S193Y	missense_variant	Somatic	0.83	not on panel
12	1368	TP53	p.A83V	missense_variant	Potentially inherited	52.72	not on panel
28	*1385*	*PIK3CA*	*p.Q546P*	*missense_variant*	*Somatic*	not on panel	0.09
28	1385	NRAS	p.Q61R	missense_variant	Somatic	0.42	not on panel
28	1385	NRAS	p.G13R	missense_variant	Somatic	3.46	not on panel
48	1398	ERBB2	p.R784H	missense_variant	Potentially inherited	50.06	not on panel
48	1398	FGFR1	NA	amplification		NA	not on panel
230	1427	CDKN2A	p.R58Q	missense_variant	Somatic	0.78	not on panel
230	1427	PIK3CA	p.P539R	missense_variant	Somatic	1.87	not on panel
226	1419	TP53	p.E171G	missense_variant	Somatic	0.36	not on panel
226	1419	IDH2	p.L128L	synonymous_variant	Somatic	2.85	not on panel
226	1419	TP53	p.P151R	missense_variant	Somatic	32.02	not on panel
228	1424	TP53	p.Q167*	stop_gained	Somatic	24.08	not on panel
228	1424	FGFR1	NA	amplification		NA	not on panel

Supplementary Figures

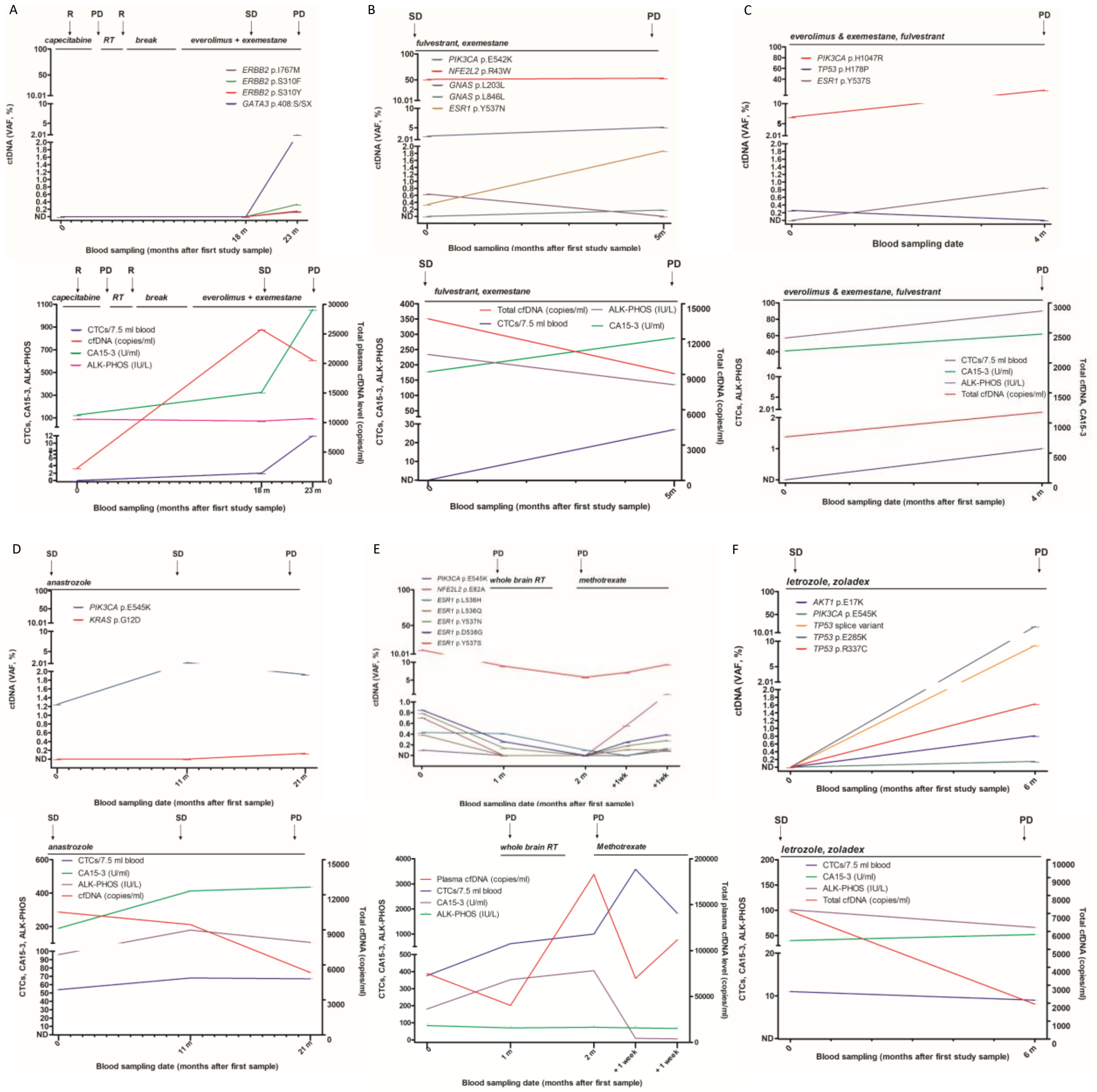

Supplementary Figure S1: Serial monitoring of alterations in ctDNA during endocrine therapy. Data from 6 patients (S1A-S1F) with mutations detectable in ctDNA tracked during clinical progression. Variant allele fractions are shown as determined by sequencing. Bottom graphs demonstrate total cfDNA concentrations (copies/mL), number of CTCs (per 7.5 ml of blood), CA15-3 (U/mL) and ALK-PHOS (IU/mL) for the same time points. Treatments details are given above each graph. SD: stable disease, PD: progressing disease, R: relapse, RT: radiotherapy, VAF: variant allele frequency, CTC: circulating tumour cell, CA15-3: Cancer antigen 15-3, ALK-PHOS: Alkaline Phosphatase
